# Biomedical engineering and ethics: reflections on medical devices and PPE during the first wave of COVID-19

**DOI:** 10.1186/s12910-021-00697-1

**Published:** 2021-09-25

**Authors:** Alessia Maccaro, Davide Piaggio, Concetta Anna Dodaro, Leandro Pecchia

**Affiliations:** 1 School of Engineering, University of Warwick, Coventry, CV47AL UK; 2 Institute of Advanced Study, University of Warwick, Coventry, CV47AL UK; 3 Department of Advanced Biomedical Sciences, University of Naples Federico II, Naples, Italy; 4 European Alliance of Medical and Biological Engineering and Science (EAMBES), Leuven, Belgium; 5 IUPESM, York, UK

**Keywords:** COVID-19, Biomedical engineering, Ethics, PPE, Regulations, Responsibility

## Abstract

In March 2019, the World Health Organization (WHO) declared that humanity was entering a global pandemic phase. This unforeseen situation caught everyone unprepared and had a major impact on several professional categories that found themselves facing important ethical dilemmas. The article revolves around the category of biomedical and clinical engineers, which were among those most involved in dealing with and finding solutions to the pandemic. In hindsight, the major issues brought to the attention of biomedical engineers have raised important ethical implications, such as the allocation of resources, the responsibilities of science and the inadequacy and non-universality of the norms and regulations on biomedical devices and personal protective equipment. These issues, analyzed one year after the first wave of the pandemic, come together in the appeal for responsibility for thought, action and, sometimes, even silence. This highlights the importance of interdisciplinarity and the definitive collapse of the Cartesian fragmentation of knowledge, calling for the creation of more fora, where this kind of discussions can be promoted.

## Background

Since early 2020, the current SARS-CoV-2 pandemic has challenged all the fields of knowledge, increasing the need for their interconnection. Medicine, science, politics, and more specialized sectors such as biomedical engineering (BME), faced crucial ethical issues, which can no longer be underestimated.

Biomedical engineers design medical devices, raising many ethical dilemmas in ordinary times, which become compelling during such a crisis. The authors of this manuscript had the privilege of different points of view thanks to the President of the European Society of BME (i.e., EAMBES) and Secretary General of the global society of BME and medical physics (IUPESM).

Three pivotal themes emerged in the global BME community:

1. The dilemma of identifying criteria for the allocation of medical devices
2. Responsibilities of science and technology
3. Inadequacy of regulations and norms, which lack universality

This manuscript does not follow the traditional structure of scientific papers (e.g., methods, results etc.), rather it is a critical analysis, revolving around the 3 above-mentioned pillars. The first pillar focuses on the surfacing of ethical dilemmas in times of pandemic (e.g., scarce resource allocation), delving into examples from Italy, where the new decision-making criteria often clashed against the existing constitutional and moral principles during the first wave.

The second pillar retrospectively reports how the medical device sector was affected by the current pandemic, touching on hazardous amatorial attempts of the general public to face the scarcity of resources and the urgent needs, and on the crisis-related challenges that surfaced for manufacturers, exacerbated by the lack of dialogue with decision-makers. In the same pillar, the theoretical debate among science and politics is addressed, referring the case of the “intended use” of a medical device, from two different philosophical perspectives, i.e., substantialism and utilitarianism. The former underlines the fundamentality of substances as ontological categories [1], suggesting that the intended use of a medical device should always be respected. The latter stresses the importance of maximizing the overall good, authorizing different uses, depending on the relativistic utility or, in exceptional times of crisis, on the emergency.

This invites the reader to a subsequent reflection on the inadequacy of the existing regulations on medical devices, in the third pillar. In respect to this, the manuscript proposes to offer a hermeneutic perspective close to the situational ethics that authorizes negotiations and mediations between the generality of principles and norms, and the specificity of the context.

Overall, the ethical considerations made in this manuscript, should be considered a valuable lesson for the future of crisis management. If and only if ethics and bioethics will be considered as effective support for science and scientists (doctors, biomedical engineers, etc.), the Cartesian separation of knowledge [2] could be overcome, establishing an interdisciplinary dialogue that involves peoples and emphasizes the public relevance of such issues.

In this way, such dilemmas could be anticipated by establishing a framework that could provide guidance and appropriate methodology to address arising urgent issues, without having to resort to specialists for questions that concern everyone and need a multidisciplinary approach.

## Allocation of medical devices: clinical and ethical principles

Due to limited resources, decision-makers have had to compromise among all the potential useful interventions. Also in the past 10 years, the most public National health systems were massively privatized, resulting in a significant reduction of prevention services and a great reduction of Intensive Care Unit (ICU) beds. The rapid spread of the SARS-CoV-2 resulted in an unprecedented need of sub-intensive and ICU beds, which overcame the capacity of the most advanced national healthcare services. Within a few weeks, the available resources (i.e., medical devices, doctors, nurses) proved to be insufficient to cover the care needs of the multitude of COVID-19 patients, beyond the ordinary needs of other patients. Consequently, doctors and healthcare structures ended up pondering and making difficult ethical choices in a short time and identifying priority principles that could guide them.

National [3–6] and international [7–9] ethics committees, scientific societies [10, 11], and experts [12–15] soon expressed their opinion on the matter [16], identifying requirements that respect human dignity and fundamental ethical principles, enshrined in the Charters of both National and International Rights [17].

The case of Italy, the first country significantly affected outside China, is emblematic. According to the Italian Constitution, health is a "fundamental right of the individual" and a "collective interest" (article 32). In addition, article 2 recognizes the personalist principle and the duty of solidarity, and article 3 establishes the principle of equality. Accordingly, the law founding the Italian National Health Services (NHS) (n. 883/1978) prescribes that care must be ensured according to the principles of universality, equality and fairness.

Leveraging on these fundamental principles, the Italian National Bioethics Committee [18] remarked the need to allocate medical devices and other resources based only on a clinical criterion, and without considering criteria such as age, gender, social attributes, ethnicity, disability and costs, in compliance with the principles of "justice, equity, solidarity". The envisaged method was that of "triage in a pandemic emergency", based on what the World Health Organization defines as "preparedness" (WHO) as a premise, and on two key concepts, i.e., "clinical appropriateness" and "actuality", identified by the healthcare professional on the basis of clinical criteria [19].

This point of view, which values the person and opposes attempts of objectification in a series of “pre-established criteria” (except for the point of view of the clinician), is well expressed by J. Habermas. In an interview with the newspaper Le Monde, Habermas underlined the inadequacy, in moral terms, of an “objective quantification” of patients and insisted on the essential issue of the “recognition” of the individual: “when addressing a second person (you, you), the other person's self-determination must either be respected or denied, that is, either accepted or ignored” [20].

On the other hand, the Italian society of anesthesiology (SIAARTI) introduced the identification of an age limit for accessing intensive care in case of necessity [21]. SIAARTI’s document explained that COVID-19 created a scenario, in which criteria for accessing ICU may be needed beyond the clinical appropriateness and proportionality of care, but also in distributive justice and the appropriate allocation of limited healthcare resources, which means privileging those with the "greatest life expectancy" [22]. Even if the choice of whom to admit to treatment is a terrible reality, it must be discussed by ethicists and bioethicists to further identify solutions that support medical doctors in taking these decisions. “This is not to deny their clinical authority and responsibility, but rather to urge a commitment to give such question public relevance" so that in the future the "public health perspective" [23] to follow is clear from the beginning.

## Responsible thinking, responsible actions, responsible silence

COVID-19 created a global lack of essential medical devices (e.g., pulmonary ventilators) and personal protective equipment (PPE, such as masks, respirators) [24]. This led to an unprecedented amount of do-it-yourself (DIY) solutions, which were fomented on media worldwide. Consequently, ordinary individuals started producing PPE at home with 3D printers and everyday materials, and manufacturers converted their production facilities to develop medical devices and PPE.

Unfortunately, although very admirable, this approach is not feasible in critical sectors such as medical devices or PPE, which require postgraduate education, years of experience and deep knowledge of relevant international standards and norms, in order to ensure appropriate levels of safety, efficacy and resilience. Thus, only 7 manufacturers in the world are producing pulmonary ventilators. In fact, during a pandemic, we do not only need to ensure the usual standard of quality, but we should also consider making those devices more resilient, because, if hospitals fail, we will need to safely operationalize these devices in field hospitals, tent-like structures, and any other relevant setting. Therefore, experience, safe-by-design approaches, and the knowledge of additional standards (e.g., military standards) become relevant too.

Minimal scientific evidence exists on how harmful this DIY wave has been, but few facts can be clearly reported referencing major newspapers. In mid-March 2020, manufacturers were called upon to help to tackle medical devices and PPE crisis.^1^,^2^ Many responded, certainly moved by the noblest principle and willingness to help. Unfortunately, learning to manufacture complicated and highly regulated pulmonary ventilators cannot be done in a few weeks. By mid-April 2020, new productions of ventilators were stopped in Spain,^3^ UK suspended orders of BlueSky ventilators^4^and France followed.^5^ Once again, a virtuous example came from Italy, where the only Italian manufacturer of ventilators (i.e., Siare Engineering International Group l.t.d.) was supported by the Italian Government, which offered 25 highly specialized army engineers, by the former FIAT (now FCA), who supported producing electro-mechanic components, in addition to Ferrari providing electronic components. With this collaborative effort, Siare increased the production of top-of-the-range ventilators from 160 to 500 units per month, respecting the highest quality standards. In conclusion, the rise of useless and potentially harmful DIY approaches to PPE and medical devices could have been easily avoided at the start of the pandemic by decision-makers initially consulting with domain experts, such as biomedical and clinical engineers.

In this regard, the belief that disciplinary competence is to be sectorized and not interconnected, very often, leads to a further separation of knowledge that is detrimental to people. In fact, it could be good practice that politicians had a solid scientific background in order to legislate about scientific matters, above all if they involve public health. On the other hand, scientists should "grovel in the dirt of the city of Romulus" [25] keeping their related studies as tangible and accessible as possible, and acquire a more solid political culture and a growing awareness of their social role. After all, the relationship between science, policy-making and politics has been controversial since the dawns of civilization: people like Aristarchus of Samos (i.e., one of the fathers of an early Heliocentrism), censored by sectaries such as Cleanthes (i.e., the prince of stoics at that age), or like Socrates, accused, “censored”, and sentenced to death for being “unorthodox” by one of the most open and democratic societies of the times, the *Polis* of Athens [26], or like Galileus, who was condemned for radically opposing to the Sacred Scriptures- and Church-approved Geocentric model, are just a few stark examples. Although much progress has been made since then, there is still an ongoing debate among a Weberian distinction between science and politics and a Habermasian and Marcusean dichotomy between the technocratic and decisionist models of scientific advice to politics. Nonetheless, it is evident that we are transitioning towards ever more present democratization of science, and not without associated risks. In fact, how can this be achieved without compromising the *epistemic quality* of knowledge [27]?

In particular, the COVID-19-related debate, involving politicians, scientists (especially biomedical engineers) and ethics experts, is based on two distinct currents of thought, referring to two different philosophical matrices, i.e., *utilitarianism* and *substantialism*. In fact, some people believe that any kind of emergency-ready response that can make up for the shortage of PPE and medical devices is “better than nothing”, even at the expense of the safety and efficiency normally guaranteed by the standards. This way of thinking is in line with Utilitarianism’s conception of maximizing happiness and overall gains for all the affected individuals. However, the “better than nothing approach” is dubious and is a well-known logical fallacy, that of the relative privation [28]. This kind of fallacious way of reasoning also justifies the misuse of something that does meet the standards but was intended for completely different purposes. In fact, the intended purpose is key, at least in the world of medical devices, and it is what safeguards the manufacturers in case their products fail if they are used “off label”. In this case, the liability falls with the individual who misused the product in the first place [29]. This concept is well portrayed by what happened in Harrow,^6^^,^^7^ where some nurses, after denouncing their precarious working conditions and the lack of PPE in the fight against COVID-19, had started using bin bags as a replacement of the unavailable PPE. In this case, the beneficial objective was given priority and the collective benefit was maximised. However, also the risk to the wearer had rapidly increased to the extent that it was not possible to predict its consequences (even negative).

Conversely, other people believe that the intended purpose of an object should be respected, in line with Substantialism’s theories, which attribute absolute value to an idea. According to this perspective, for example, a bin bag would be designed, tested and marketed to contain rubbish, not to protect healthcare workers from diseases (in this case the design principles and the tests will be different and stricter). Thus, these people tend towards a minimisation of the risk, but, at the same time, their precautional approach hinders the possible benefit underlying the other “less safe” alternatives. In this regard, it is necessary to recall the philosophy of Hans Jonas who, faced with an indeterminate and potential risk, the consequences of which cannot be estimated, introduced the *imperative of responsibility* in defence of future generations and based on the precautionary principle. His "heuristics of fear" implies foresight and ability to predict and adequately assess the consequences of collective activities in contemporary societies. Such principle implies to “act so that the effects of your action are compatible with the permanence of genuine life” and, in our present choices, to “include the future wholeness of Man among the objects of your (our) will” [30].

Consequently, this responsibility goes beyond the personal one of engineers, as it also includes the responsibility they partially assume if and when they do not limit reckless or inadequately considered actions, guided by the above-mentioned utilitarian approach. In fact, the compliance with international standards and the consequent CE marking does not only guarantee the quality, safety, efficiency, and efficacy of a product, but also the protection of manufacturers and users. In fact, as afore-mentioned from a general point of view, those to be blamed for the possible failure of bin bags used as PPE for the prevention of COVID-19 and the consequent infection and potential death of the healthcare workers using them are not the manufacturers of such items, but whomever decided to use this amateur substitution to other certified means of protection, and, to a certain extent, the biomedical engineers who did not respect their duty to identify the limits regarding the unintended uses.

Overall, two theoretical orientations are at the basis of these dichotomous approaches. However, in order to better frame them, it is necessary to analyse them in view of the extraordinary condition of necessity begotten by the pandemic. The dilemma revolves around the "intended use", or rather the purpose for which something (e.g., PPE or medical devices) was originally designed for: on the one hand there are those who assert that, in conditions of necessity, the contingent purpose, i.e., the social functionality that overcomes the intended use, ought to be preferred despite being “off label”. In fact, they firmly believe that it is preferable to maximize the current benefit while assuming an undefined risk. Although it is not easy to relate this trend to a specific current of thought, it certainly shares some points in common with utilitarianism, starting from the Benthamian one [31], if not with pragmatism (e.g., Dewey [32]).

On the other hand, the position of those who consider the “intended use” or rather the intrinsic purpose for which the product was manufactured tested and marketed, a priority, would seem evocative of Aristotelian substantialism or eschatology. In this case, the risks are limited by compliance with the law and the relevant standards, which also guarantee the achievement of the benefits. The refusal of this immediately relieves the manufacturer and the regulator from any responsibility related to the misuse of the object, leaving every possible and unforeseeable risk to the individual.

Here lies the crux of the problem. Using what is available and certified, albeit designed for a different “intended use”, seems more “reasonable” than not protecting oneself to everyone. However, we cannot refrain from asking ourselves the following questions: what is the limit within which it is possible to say, “better than nothing”? To what extent can science and policymakers put people's lives at risk in order to have a prompt, but probably unsafe answer in the wake of the "better than nothing" principle?

Regulatory frameworks and standards should be reviewed in this regard.

Beyond the DIY solutions, low-quality outputs have been affecting scientific production too. In fact, the high demand for information caused an acceleration in reporting scientific results, with many journals being overwhelmed with unprecedented numbers of papers, which challenged the capability of editors and reviewers to scrutinise articles.

The unprecedented high number of retracted papers can be a proxy for the high number of low-quality research on COVID-19. For this reason, a rapid search for papers regarding COVID-19 or SARS-CoV-2, and the previous epidemic/pandemics (i.e., SARS, MERS, Swine Flu) as a comparison, was performed both on OvidSP and the Retraction Watch Database. As regards COVID-19 publications, there were 124 retracted papers out of 264,530, i.e., 4.68 per 10,000 papers (compared to 1.16 per 10,000 papers concerning the previous pandemics/epidemics). Although this proxy is to be taken with a grain of salt, it should be a wake-up call for further investigations. Similar levels of confusion could be observed also among scientists and experts invited by media to interpret available scientific evidence and technical guidance.

The above-mentioned issues contributed to beget and feed an *infodemic*, defined by the United Nation as “an over-abundance of information—some accurate and some not—that makes it hard for people to find trustworthy sources and reliable guidance when they need it” [33], which is inducing an unprecedented need for responsible silence too.

## Inadequacy of regulatory frameworks and norms

The pandemic creates a generalized condition of resource limited settings (RLSs), i.e., environments lacking means, specific knowledge, specialized personnel, medical devices and drugs within inappropriate medical locations. While this condition was already familiar to low- and middle-income countries, COVID-19 has overwhelmingly created RLS conditions in high-income countries, such as Europe, the USA and Japan, for the first time since World War II. This demonstrates how regulatory frameworks for medical devices and PPE are inadequate to RLS conditions. In fact, these regulations usually take into consideration standards that are too stringent and generic, proving impossible to fulfil in RLSs and, in times of the pandemic, difficult to adhere to universally. For example, the numerous tests and verifications required to assess the conformity of market respiratory protective equipment or eye protection equipment for healthcare purposes wasted time. One reason for this is that these standards are influenced by big manufacturers interested in having the largest market share. As a result, commercial standards for PPEs require testing in conditions that are not relevant for hospital workers (e.g., high temperature typical of heavy metals industry). Hence, international standards and norms followed the principle of generalism, losing universality and creating unnecessary burdens for small manufacturers [34]. In this regard, the WHO has published, for the first time, technical guidance on PPE specifically relevant for healthcare settings [35, 36]. Differently to ISO standards for masks and respirators, the WHO guidance focuses on essential parameters, such as filtering capability, fit and breathability for masks.

Recalling the two aforementioned ethical perspectives, considering existing medical device regulation too generic to be universal, does not mean adhering to the utilitarian-pragmatic current tout court. There may be a contextualised response, regulated on the basis of tests, complying with flexible standards, or rather standards that are purposely designed to take into account different niche conditions. However, the use of any object must be certified and not random, and subject to tests relating to its specific intended use. Only in this way, people and their rights can be safeguarded, and science can prove to think deeply, act consciously and remain silent, when appropriate.

Contextualism is the basis of situational ethics [37], which seems to be the most adequate response to the specific needs of everyone and be able to face emergencies. In fact, it starts from the particular situation and tries to find universalizable answers, applying a heuristic and inductive method, progressive negotiations and interdisciplinary exchanges.

## Conclusions

One year after the start of the pandemic, the need for ethic guidance is still tangible in everyday circumstances and essential during crisis or in RLSs. Respecting fundamental ethical principles while negotiating among different criteria (hospitalization demands vs available ICUs, generalism vs particularism, action vs responsible-action) requires clear guidance, deep knowledge, and peer-to-peer discussion among experts of different disciplines. The need for extreme specialization should never result in the fragmentation of knowledge.

Exactly a century, i.e., the *Short Twentieth Century*, separates COVID-19 from the last pandemic, the so-called "Spanish Flu", which flagellated Europe in 1918–1919. According to Hobsbawm, “*no period in history has been more penetrated by and more dependent on the natural sciences*” and “*yet no period, since Galileo's recantation, has been less at ease with it*”. This chasm between scientists and the general public is still open and, in some cases, fomented by populisms, which leverage on people's fears evoking war atmospheres, which have nothing to do with the catastrophic failure of many national healthcare systems’ response to this crisis. After a century, the dependence of medicine on biomedical science and engineering is evident, while their contribution to the definition of effective policies and norms is still negligible. Finally, the Cartesian fragmentation of knowledge, or rather “thinking in silos”, has persisted across the last century, calling for the creation of more fora where multidisciplinary discussions can be promoted. Three main needs emerged clearly: the need for responsible thinking, the need for responsible action and the need for responsible silence, when required and appropriate [38].

## Data Availability

Not applicable.

## Abbreviations

WHO: World Health Organization
BME: Biomedical Engineering
EAMBES: European Alliance for Medical and Biological Engineering & Science
IUPESM: International Union for Physical and Engineering Sciences in Medicine
ICU: Intensive Care Unit
SIAARTI: Società Italiana di Anestesia Analgesia Rianimazione e Terapia Intensiva
NHS: National Health Service
PPE: Personal Protective Equipment

## Footnotes

1 https://www.ft.com/content/491a4af4-66e7-11ea-a3c9-1fe6fedcca75.

2 https://www.nytimes.com/2020/03/20/us/politics/trump-coronavirus-supplies.html.

3 https://www.lavanguardia.com/vida/20200411/48413026980/seat-detiene-produccion-respidradores-emergencia-descongestion-uci.html.

4 https://uk-mobile-reuters-com.cdn.ampproject.org/c/s/uk.mobile.reuters.com/article/amp/idUKKCN21U0UI.

5 https://www.franceinter.fr/coronavirus-8-500-respirateurs-produits-pour-rien.

6 https://www.bbc.co.uk/news/health-52145140.

7 https://www.telegraph.co.uk/news/2020/04/08/exclusive-three-nurses-forced-wear-bin-bags-ppe-shortage-test/.

## References

[1] French S (2018). Between factualism and substantialism: Structuralism as a third way. Int J Philos Stud.

[2] Morin E (1992). From the concept of system to the paradigm of complexity. J Soc Evolut Syst.

[3] Department for Health of Ireland. Ethical framework for decision-making in a pandemic. https://assets.gov.ie/72072/989943ddd0774e7aa1c01cc9d428b159.pdf (2020). Accessed 19 June 2020.

[4] The Nuffield Council of Bioethics. Responding to the COVID-19 Pandemic: Ethical Considerations. https://www.nuffieldbioethics.org/news/responding-to-the-covid-19-pandemic-ethical-considerations (2020). Accessed 19 June 2020.

[5] Comité De Bioética De España. Informe Del Comité De Bioética De España Sobre Los Aspectos Bioéticos De La Priorización De Recursos Sanitarios En El Contexto De La Crisis Del Coronavirus. http://assets.comitedebioetica.es/files/documentacion/Informe CBE-Priorizacion de recursos sanitarios-coronavirus CBE.pdf (2020). Accessed 19 June 2020.

[6] Deutscher Ethikrat. Solidarity and responsibility during the coronavirus crisis. https://www.ethikrat.org/en/press-releases/2020/solidarity-and-responsibility-during-the-coronavirus-crisis/ (2020). Accessed 19 June 2020.

[7] UNESCO. Ethics in Research in Times of Pandemic COVID-19. https://en.unesco.org/news/ethics-research-times-pandemic-covid-19 (2020). Accessed 19 June 2020.

[8] UNESCO. Statement on COVID-19: ethical considerations from a global perspective. https://unesdoc.unesco.org/ark:/48223/pf0000373115 (2020). Accessed 19 June 2020.

[9] WHO. Ethical standards for research during public health emergencies: distilling existing guidance to support COVID-19 R&D. https://www.who.int/blueprint/priority-diseases/key-action/liverecovery-save-of-ethical-standards-for-research-during-public-health-emergencies.pdf?ua=1 (2020). Accessed 19 June 2020.

[10] Berlinger N, Wynia M, Powell T, Hester DM, Milliken A, Fabi R, et al. Ethical framework for health care institutions responding to novel Coronavirus SARS-CoV-2 (COVID-19) guidelines for institutional ethics services responding to COVID-19. Safeguarding communities, guiding practice, 2; 2020.

[11] Meyfroidt G, Vlieghe E, Biston P, De Decker K, Wittebole X, Collin V, et al. Ethical principles concerning proportionality of critical care during the COVID-19 pandemic: advice by the Belgian Society of IC medicine. Retrieved 2 April 2020. https://www.hartcentrumhasselt.be/professioneel (2020).

[12] Emanuel EJ, Persad G, Upshur R, Thome B, Parker M, Glickman A (2020). Fair allocation of scarce medical resources in the time of Covid-19.

[13] Smith MJ, Ahmad A, Arawi T, Dawson A, Emanuel EJ, Garani-Papadatos T (2021). Top five ethical lessons of COVID-19 that the world must learn. Wellcome Open Res.

[14] Smith MJ, Upshur RE (2020). Learning lessons from COVID-19 requires recognizing moral failures. J Bioeth Inq.

[15] Smith MJ, Upshur RE, Emanuel EJ (2020). Publication ethics during public health emergencies such as the COVID-19 pandemic. Am J Public Health.

[16] WHO. Resources on ethics and COVID-19. https://www.who.int/ethics/topics/outbreaks-emergencies/covid-19/en/ (2020). Accessed 19 June 2020.

[17] WHO. Addressing human rights as key to the COVID-19 response. https://www.who.int/publications-detail/addressing-human-rights-as-key-to-the-covid-19-response (2020). Accessed 19 June 2020.

[18] Comitato nazionale per la bioetica. Covid 19: clinical decision-making in conditions of resource shortage and the "pandemic emergency triage criterion". http://bioetica.governo.it/media/3987/p136_2020_covid-19-la-decisione-clinica-in-condizioni-di-carenza-di-risorse-e-il-criterio-del-triage-in-emergenza-pandemica.pdf (2020). Accessed 19 June 2020.

[19] WHO Preparedness. https://www.who.int/environmental_health_emergencies/preparedness/en/ (2020). Accessed 19 June 2020.

[20] The only cure is solidarity from Jürgen Habermas. https://www.classlifestyle.com/news/42575/kura-e-vetme-eshte-solidariteti-nga-jrgen-habermas/eng (2020). Accessed 19 June 2020.

[21] Vergano M, Bertolini G, Giannini A, Gristina GR, Livigni S, Mistraletti G (2020). Clinical ethics recommendations for the allocation of intensive care treatments in exceptional, resource-limited circumstances: the Italian perspective during the COVID-19 epidemic.

[22] Rosenbaum L (2020). Facing COVID-19 in Italy—ethics, logistics, and therapeutics on the epidemic’s front line. N Engl J Med.

[23] Nicoli F, Gasparetto A (2020). Italy in a time of emergency and scarce resources: the need for embedding ethical reflection in social and clinical settings. J Clin Ethics.

[24] Ranney ML, Griffeth V, Jha AK (2020). Critical supply shortages—the need for ventilators and personal protective equipment during the COVID-19 pandemic. N Engl J Med.

[25] Sokolov P, Ivanova J. 17th century political cartesianism and its opponents, or imaging the state from point fixe. Higher School of Economics Research Paper No. BRP, 8. 2012.

[26] Jones D (2001). Censorship: a world encyclopedia.

[27] Maasen S, Weingart P (2006). Democratization of expertise? Exploring novel forms of scientific advice in political decision-making.

[28] Bennett B. Logically fallacious: the ultimate collection of over 300 logical fallacies (Academic Edition). EBookIt.com. 2017.

[29] Regulation EU 2017/745 of the European Parliament and of The Council of 5 April 2017 on medical devices, amending Directive 2001/83/EC, Regulation (EC) No 178/2002 and Regulation (EC) No 1223/2009 and repealing Council Directives 90/385/EEC and 93/42/EEC (2017). In: The European parliament and the council of the European Union (Ed.).

[30] Jonas H. The imperative of responsibility: In search of an ethics for the technological age. 1984.

[31] Bentham J, Mill JS (2004). Utilitarianism and other essays.

[32] Dewey J (1998). The essential Dewey: pragmatism, education, democracy.

[33] WHO. Novel coronavirus (2019-nCoV) Situation Report-13; 2020.

[34] Pecchia L, Piaggio D, Maccaro A, Formisano C, Iadanza E (2020). The inadequacy of regulatory frameworks in time of crisis and in low-resource settings: personal protective equipment and COVID-19. Health Technol.

[35] WHO. Rational use of personal protective equipment for COVID-19 and considerations during severe shortages: interim guidance, 23 December 2020. World Health Organization; 2020.

[36] WHO. Technical specifications of personal protective equipment for COVID-19.

[37] Fletcher JF (1997). Situation ethics: the new morality.

[38] Maccaro A, Piaggio D, Pagliara S, Pecchia L (2021). The role of ethics in science: a systematic literature review from the first wave of COVID-19. Health Technol..

